# Electric Transport in Gold-Covered Sodium–Alginate Free-Standing Foils

**DOI:** 10.3390/nano11030565

**Published:** 2021-02-24

**Authors:** Carlo Barone, Monica Bertoldo, Raffaella Capelli, Franco Dinelli, Piera Maccagnani, Nadia Martucciello, Costantino Mauro, Sergio Pagano

**Affiliations:** 1 Dipartimento di Fisica “E.R. Caianiello”, Università degli Studi di Salerno, I-84084 Fisciano, Italy; ing.costantinomauro@gmail.com; 2 CNR—SPIN Salerno, c/o Università degli Studi di Salerno, I-84084 Fisciano, Italy; nadia.martucciello@spin.cnr.it; 3 INFN Gruppo Collegato di Salerno, c/o Università degli Studi di Salerno, I-84084 Fisciano, Italy; 4 Dipartimento di Scienze Chimiche, Farmaceutiche ed Agrarie, Università degli Studi di Ferrara, Via L. Borsari 46, I-44121 Ferrara, Italy; brtmnc@unife.it; 5 Istituto per la Sintesi Organica e la Fotoreattività, Consiglio Nazionale delle Ricerche, Via P. Gobetti 101, I-40129 Bologna, Italy; 6 Dipartimento di Ingegneria E. Ferrari, Università di Modena e Reggio Emilia, I-41125 Modena, Italy; capelli@unimore.it; 7 CNR—Istituto Officina dei Materiali, S.S. 14, km 163.5 in Area Science Park, I-34012 Trieste, Italy; 8 Department of Physics, University of Johannesburg, P.O. Box 524, Auckland Park 2006, South Africa; 9 CNR—Istituto Nazionale di Ottica, Via G. Moruzzi 1, I-56124 Pisa, Italy; franco.dinelli@ino.cnr.it; 10 CNR—Istituto per la Microelettronica e Microsistemi, Via P. Gobetti 101, I-40129 Bologna, Italy; maccagnani@bo.imm.cnr.it

**Keywords:** biopolymers, electric transport measurements, gold thin films

## Abstract

The electric transport properties of flexible and transparent conducting bilayers, realized by sputtering ultrathin gold nanometric layers on sodium–alginate free-standing films, were studied. The reported results cover a range of temperatures from 3 to 300 K. In the case of gold layer thicknesses larger than 5 nm, a typical metallic behavior was observed. Conversely, for a gold thickness of 4.5 nm, an unusual resistance temperature dependence was found. The dominant transport mechanism below 70 K was identified as a fluctuation-induced tunneling process. This indicates that the conductive region is not continuous but is formed by gold clusters embedded in the polymeric matrix. Above 70 K, instead, the data can be interpreted using a phenomenological model, which assumes an anomalous expansion of the conductive region upon decreasing the temperature, in the range from 300 to 200 K. The approach herein adopted, complemented with other characterizations, can provide useful information for the development of innovative and green optoelectronics.

## 1. Introduction

One of the most relevant and emerging fields of research in recent years concerns sustainability, with particular reference to the significant amount of energy consumed for realizing and powering the electronic components during their lifetime [1,2,3]. In this respect, it is known that conventional electronics, which are based on natural elements and individual components, have costs, including their recycling, that can be so high as to become inconvenient in many cases [4,5]. Therefore, the scientific community is increasingly focusing on the realization of innovative devices which have reduced production energy and disposal expenses (“green electronics”).

Within this area, renewable polymeric materials derived from nature have gained great popularity due to their excellent processability, mechanical properties, and recycling efficiency [6,7,8,9]. Among them, sodium alginate (SA), a natural biopolymer extracted from marine algae, presents important characteristics of non-toxicity, biocompatibility, and biodegradability [10]. Due to its transparency, good protonic conductivity, and film-forming adaptability, SA is expected to find useful applications in green electronic devices [11,12]. However, only a very few studies have been reported in the scientific literature on this topic. Therefore, a detailed investigation of the possibility of integrating SA films into electronic circuitry is necessary, starting from the realization of highly conductive contacts [13]. To this scope, a crucial aspect regards the process of metallization and, as a consequence, the interface properties of the metallic layers with the organic compounds [14].

Different techniques can be adopted to obtain a metallization, such as electrochemical or chemical vapor deposition, sputtering or thermal evaporation, and finally non-covalent functionalization, in which the assembly of the pristine species is mildly carried out in water at room temperature [15]. Moreover, the choice of a metal in connection with a polymer has a strong influence on the physical and chemical final properties of the conductive films [16]. At present, electrochemical deposition is one of the most interesting preparation methods and the use of silver nanoparticles or nanowires in nanopapers based on nanocellulose represents one of the most promising technologies [8,11,12]. However, electrochemical deposition is not suitable for the realization of ultrathin (<10 nm) transparent and conductive layers, requested, for example, in the case of optoelectronic applications. In addition, the fabrication costs of pure nanopapers are relatively high. Therefore, the development of alternative solutions is a relevant topic in green electronics.

A different approach, consisting of sputtering gold onto SA free-standing substrates, has been recently proposed by some of the authors. Herein, a detailed study of the electric transport properties of these bilayers was performed in the temperature range from 3 to 300 K. SA is inexpensive and can be easily manipulated. Furthermore, the proposed metallization process allows one to deposit ultrathin gold layers, maintaining a smooth morphology, high mechanical stability, and good transparency [3,14]. Despite the large amount of structural, mechanical, and optical characterizations reported in the literature on alginate compounds [3,14,17,18], few studies of their electric transport properties, if any at all, are presently known. Temperature-dependent transport measurements can provide useful information on the physics of the electrical conduction mechanisms, as demonstrated in the case of superconducting and magnetic materials [19,20,21,22], granular aluminum oxide thin films [23,24,25], and oxide interfaces [26,27,28]. The results obtained can be very useful from a technological point of view. In particular, they show the feasibility of realizing organic conducting devices that can also operate in a cryogenic environment, using natural polymer films and metallic layers of a nanometric thickness.

## 2. Experimental Results and Discussion

**Figure 1:**
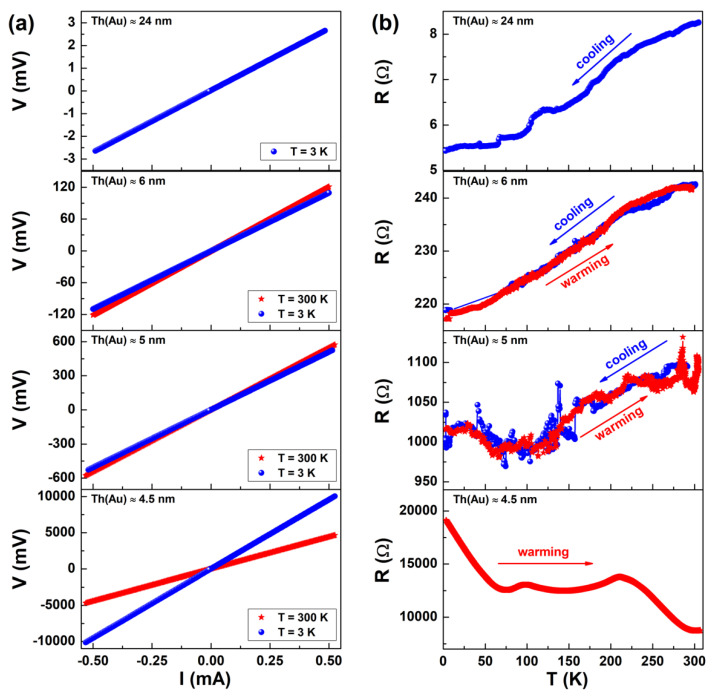
DC electrical characterization of four sodium–alginate (SA) samples sputtered with Au, with a thickness ranging from 4.5 to 24 nm. (**a**) Current–voltage (I-V) curves. Red stars data refer to 300 K, while blue circles refer to 3 K. (**b**) Resistance versus temperature (R-T) curves are reported for the same samples. The curves, acquired in cooling (blue circles) and warming (red stars) modes, did not show any significant hysteresis.

A detailed DC electric transport investigation was performed from 3 to 300 K on four SA films, covered with thin sputtered gold (Au) layers of different thicknesses. As shown in Figure 1a (left panel), all the current–voltage (I-V) curves were linear; that is, all the samples had an ohmic behavior, regardless of both the Au thickness and the temperature at which the measurements were performed: 300 K (red stars) or 3 K (blue circles). Conversely, plotting resistance versus temperature (R-T), a strong dependence of R on the Au thickness can be found, as displayed in Figure 1b (right panel). In particular, it can be noticed that R increased with increasing T, thus exhibiting a “metallic” behavior for thicknesses of 24 and 6 nm (first and second graphs from the top). A less pronounced “metallic” behavior can be observed for a thickness of 5 nm (third graph), while an increase in R upon lowering T was found for a thickness of 4.5 nm (fourth graph). From the point of view of the electrical transport, this last case is the most useful for the comprehension of the mechanisms involved in the intrinsic electrical response of the SA free-standing foils coated with nanometric films of sputtered Au.

**Figure 2:**
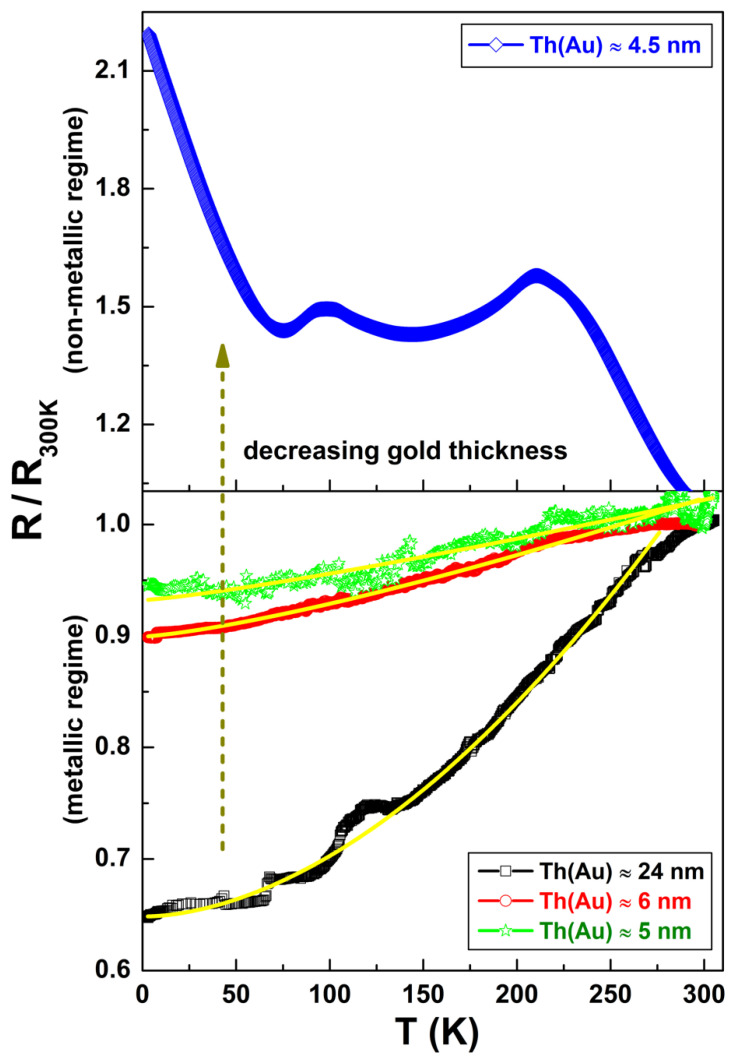
Temperature dependence of the ratio $R/R_{300K}$, where $R_{300K}$ is the room temperature resistance value. From an Au layer of 24 nm (black squares) down to 6 nm (red circles) and 5 nm (green stars), the behavior became increasingly less metallic (lower panel). A change in the electrical transport mechanism is clearly visible for the thinnest Au film (blue diamonds), characterized by a strong non-metallic behavior (upper panel).

The effect related to the Au thickness was more evident if the R values are plotted, normalizing the data to the room temperature resistance $R_{300K}$. The results are visible in Figure 2, where the T dependencies of the ratio $R/R_{300K}$ are shown, evidencing a clear reduction in the metallicity of the samples upon decreasing the Au thickness. These experimental data can be analyzed with a power-law expression [29]:

(1) $$\frac{R\left(T\right)}{R_{300K}}\;=\;R_{0}+R_{1}T^{n},\;$$

where $R_{0}$ is the low-temperature residual normalized resistance, $R_{1}$ is a multiplicative factor, and n is an exponent, which depends on the nature of the carrier interactions considered. The best-fit procedure using Equation (1), yellow lines in the lower panel of Figure 2, yielded the following values: n = 1.8 ± 0.2 for an Au thickness of 24 nm, n = 1.3 ± 0.1 for 6 nm, and n = 1.2 ± 0.1 for 5 nm. This analysis indicates that the thickest film is characterized by a standard metallic Fermi-liquid behavior, corresponding to n = 2 [29,30,31]. Charged impurity resistance contributions become dominant for thinner Au films (5 and 6 nm), corresponding to n = 1 [32,33]. Similar results have been observed in the case of thin epitaxial Au films deposited on sapphire [34] and also for ultrathin Au films grown on other transparent polymers [35]. A strong increase in the resistivity values was also correlated to the reduction in the metal thickness, as shown in Figure 2. This behavior can be ascribed to an enhanced surface scattering of the conduction electrons and structural defects, additionally built-in during the initial stages of the sputtering process [34]. The presence of these intrinsic defects and dislocations contributes to the formation of electrically isolated clusters in the thinner films, while thicker samples are highly structured [35]. The possible consequence of these morphological properties, characteristic of the Au growth, could be the occurrence of a crossover from a typical Fermi-liquid behavior to a less “metallic” one upon decreasing the Au thickness. This corresponds to the experimental results shown in Figure 2 and to the outcome of the modeling.

A noticeable change in the R-T dependence was, instead, found for an Au thickness of 4.5 nm. As evidenced in the upper panel of Figure 2, the metallic behavior was not observed anymore (blue diamonds), in agreement with the experimental observations reported for ultrathin Au nanostructures sputtered on glass [36]. For those structures, upon lowering the layer thickness, it has been observed a lattice expansion, which is manifested in an increase in the lattice parameter and, consequently, a decrease in the metal density [37,38,39,40].

**Figure 3:**
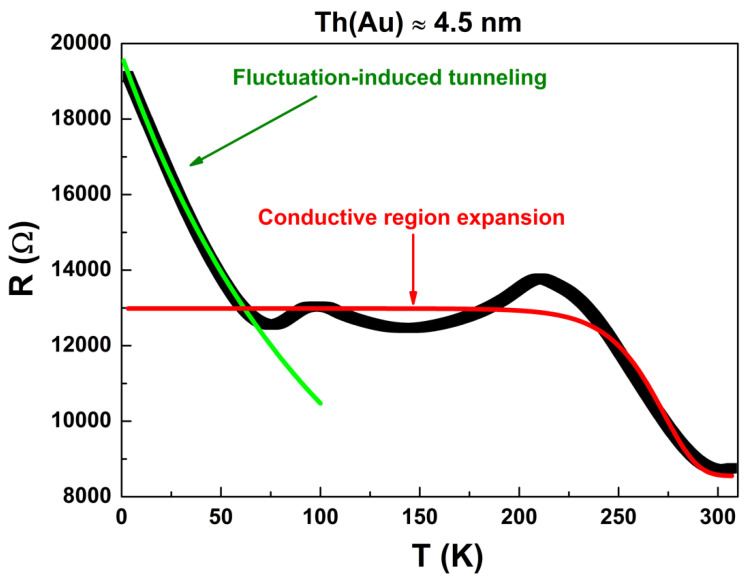
Modeling of the DC experimental data obtained for the thinnest Au film (4.5 nm). The R-T dependence (black dots) could be reproduced with the fluctuation-induced tunneling model below 70 K (green curve, best fit using Equation (2)) and with a phenomenological model that assumes an expansion of the conductive region from 300 to 200 K (red curve, best fit using Equation (4)).

The low-temperature regime, below 70 K, was characterized by a net R increase upon decreasing T. To understand this behavior, the disordered nature of the random resistor network constituting the structure of the ultrathin Au film has to be considered. In this framework, a suitable description of the electrical transport mechanisms is given by the fluctuation-induced tunneling (FIT) model [41,42,43]. According to this model, the electrical conduction is dominated by electron transfer between large conducting segments rather than by hopping between localized sites. As a consequence, the electrons tend to tunnel between conducting regions at the points of their closest contact, where the relevant tunnel junctions are usually small in size and, therefore, are exposed to large thermally activated voltage fluctuations [41]. Following this model, the R-T dependence can be expressed as:

(2) $$R\left(T\right)\;=\;R_{FIT}exp\left[\frac{T_{1}}{T+T_{0}}\right],\;$$

where $R_{FIT}$ is a preexponential factor, $T_{0}$ and $T_{1}$ are two characteristic temperatures of the system investigated. The applicability of this model has been shown, for example, in the case of magnetic materials [44,45,46] and of carbon nanotube composites [47,48,49]. The solid green line, shown in Figure 3, was obtained from Equation (2) with the best fitting value of $T_{0}\;=\;\left(90\pm 7\right)$ K. This value gives a direct estimation of the temperature below which tunneling between the conducting regions becomes significant. From the best fit procedure, a value of $T_{1}\;=\;\left(1010\pm 70\right)$ K was obtained and, being related to the energetic scale of the formed insulating barriers, is in agreement with what already found in the case of disordered systems [41].

Above 70 K, instead, the curve substantially flattened out with some oscillations. This region was then followed by a reduction in R for increasing T. To explain this behavior, a phenomenological model was considered that assesses an expansion of the conductive region upon decreasing T. This expansion, which would be anomalous for a homogeneous material, can, however, be possible if one considers that the conductive layer can be described as a dense collection of clusters embedded in the biopolymer film, which contains an unknown amount of water molecules.

The average distance between the clusters can be expressed in terms of a general exponential function as:

(3) $$a\left(T\right)\;=\;c_{0}-c_{1}exp\left[c_{2}\left(T-T^{⋆}\right)\right].\;$$

Here, $c_{0}$, $c_{1}$, and $c_{2}$ are free fitting parameters, and $T^{⋆}$ is fixed to the value of 273 K. The contribution to the total R can be consequently ascribed to a rectangular tunneling barrier with a width a(T). Then, it is straightforward to derive the following expression for R versus T:

(4) $$R\left(T\right)\;=\;R_{M}+R_{EXP}exp\left[a\left(T\right)\right],\;$$

where $R_{M}$ is a high-temperature constant resistance term, probably due to the conducting paths originated by the ohmic Au connections, and $R_{EXP}$ is a preexponential factor. The best fit procedure with Equation (4) gave an appreciable agreement, and is also statistically consistent, with the experimental data in the region from 300 down to 70 K. This is shown in Figure 3 with the solid red line.

Overall, the investigation of a wide range of temperature values is a decisive approach to describe the electric transport processes at work in Au sputtered SA structures appropriately. A more detailed understanding of the effect due to the polymeric matrix on the conduction could be achieved through the realization and the analysis of new samples prepared under different conditions. This will be the object of future investigations.

## 3. Materials and Methods

Alginic Acid Sodium Salt (SA) was purchased from Sigma–Aldrich (Milano, Italy) and solubilized in ultrapure water at room temperature. A quantity of 32 mL of a solution at 2% wt. concentration was cast in a 100 mm polystyrene Petri dish. After drying at room temperature for several days, transparent free-standing films were obtained. A thin Au layer was sputtered onto the SA films using an MRC 8622 RF system (Kenosistec s.r.l, Binasco, Milano, Italy). The deposition process was performed at low power (20 W) to avoid unwanted substrate heating and to have a finer control of the thickness. The complex morphology of these films made it difficult to evaluate the value of their thickness directly. A calibration was then performed using as reference a flat piece of silicon wafer placed in the chamber beside the SA film when depositing 24 nm of Au. The nominal deposition rate was calculated to be 0.05 nm s^−1^.

**Figure 4:**
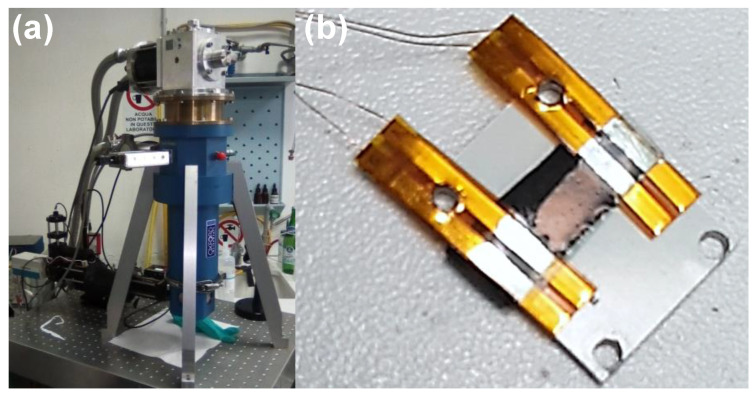
Experimental setup components. (**a**) Photograph of the closed-cycle refrigerator used for the electrical measurements as a function of the temperature. (**b**) Photograph of the sample holder used.

The electrical properties of the samples were characterized using a dedicated setup. The temperature control was obtained with a closed-cycle refrigerator, mod. CSW-71 compressor and RDK-408D cold-head (Sumitomo (SHI) Cryogenics of Europe GmbH, Darmstadt, Germany), capable of reaching a base temperature of 3 K and with a 1 W refrigeration power capability at 4.2 K (see Figure 4a). Similar to what was described in [50,51,52], a low-noise electronic bias and readout circuitry was used. The readout electronics were controlled using a dedicated computer equipped with high-resolution digital to analog and analog to digital interfaces through a PXI crate (National Instruments, Austin, TX, USA) and programmed using the LabVIEW environment.

The electrical connections were made by contacting the sample with two flexible printed circuits (flexPCB), each made by two Au-covered copper strips embedded in Kapton placed at a distance of about 5 mm, to realize a four contacts configuration, see Figure 4b. The sample and flexPCB were sandwiched between two Teflon-covered Al plates and kept in position using plastic screws.

## 4. Conclusions

Starting from a natural biopolymer foil obtained from marine algae sodium–alginate, conducting films were prepared by sputtering thin gold nanolayers with a thickness variable from 4.5 to 24 nm. The electric transport properties of the samples realized were then investigated in the temperature range from 3 to 300 K.

A “metallic” behavior was observed from the temperature dependence of the film resistance for a gold thickness above 5 nm. The conducting film could also be maintained quasi-transparent, keeping the gold thickness below 6 nm.

Below 5 nm, instead, the temperature dependence of the electrical conduction was quite different. In a temperature region below 70 K, the electrical transport can be well explained in terms of fluctuation-induced tunneling through gold clusters embedded in the sodium–alginate film. Above 70 K, in particular, in the range from 200 to 300 K, a phenomenological model can be used to explain the experimental findings. Such a model assumes an increase in the average distance between the gold clusters upon decreasing the temperature.

The information extracted from the electrical analysis herein reported, correlated with other characterizations, can be very useful to investigate this type of compound. In addition, the absence of toxic components and the intrinsic biodegradability of sodium–alginate make the system investigated a real example of green technology and a very promising candidate to be employed in flexible green optoelectronics.
